# A nomogram model for preoperative evaluation of invasive degree in lung adenocarcinoma: integrating intratumoral and 2 mm peritumoral radiomics scores with conventional features

**DOI:** 10.3389/fmed.2026.1984781

**Published:** 2026-10-28

**Authors:** Minglang Yang, Yue Zhang, Jingyi Wang, Yanyan Zhang, Xiaohui Wang, Jing Wen, Baoying Zhao, Xu Dai

**Affiliations:** 1 The First Clinical College, Liaoning University of Traditional Chinese Medicine, Shenyang, Liaoning, China; 2 Department of Radiology, The Affiliated Hospital of Liaoning University of Traditional Chinese Medicine, Shenyang, Liaoning, China; 3 CT Clinical Science, Philips (China) Investment Co., Ltd., Shenyang, Liaoning, China

**Keywords:** CT, intratumoral, invasiveness, lung adenocarcinoma, peritumoral, radiomics

## Abstract

**Objective:**

To determine the optimal peritumoral margin for predicting invasiveness in lung adenocarcinoma and to develop and validate a nomogram integrating conventional CT features with intratumoral and peritumoral radiomics scores for non-invasive preoperative assessment of invasive degree.

**Methods:**

This retrospective study included 171 patients with pathologically confirmed pulmonary nodules from the Affiliated Hospital of Liaoning University of Traditional Chinese Medicine (January 2018 – December 2025), randomly split into training (*n* = 120) and test (*n* = 51) sets (7:3 ratio). Based on the 2021 WHO classification, nodules were classified as pre-microinvasive (AAH, AIS, MIA) or invasive (IAC). Conventional CT features and 107 radiomics features were extracted from intratumoral and varying peritumoral margins (0–2, 0–4, 0–6, and 0–8 mm). Feature selection was performed using LASSO and logistic regression to construct five models: a conventional model, an intratumoral radiomics score model, peritumoral radiomics score models (at different margins), an intratumoral-peritumoral radiomics score model, and a combined conventional-intratumoral-peritumoral radiomics model. Performance was assessed via ROC curves, DCA, calibration curves, nested 5-fold cross-validation (25 iterations) and nested Bootstrap (1,000 iterations). Model interpretability was analyzed using SHAP (SHapley Additive exPlanations).

**Results:**

Multivariate logistic regression identified maximum diameter and mean CT value as independent predictors. Among the four peritumoral ranges, the 2 mm peritumoral radiomics score model showed optimal performance (AUC: 0.902 training, 0.881 test), establishing 2 mm as the optimal margin. The combined model (conventional + intratumoral + 2 mm peritumoral) achieved the best predictive performance (AUC: 0.925 training, 0.888 test), outperforming the conventional and single radiomics models. The model demonstrated good calibration (Hosmer-Lemeshow test, *P* > 0.05). To prevent information leakage, nested 5-fold cross-validation (25 iterations) and nested Bootstrap (1,000 iterations) were performed on the training set, yielding a mean AUC of 0.873 ± 0.079 and 0.763 (95% CI: 0.572–0.907), respectively. DCA confirmed a favorable net clinical benefit. SHAP analysis identified the intratumoral-2 mm peritumoral radiomics score, mean CT value, and maximum diameter as the top three contributing features.

**Conclusion:**

A 2 mm peritumoral margin is optimal for predicting lung adenocarcinoma invasiveness. The proposed nomogram enables non-invasive, accurate preoperative prediction with strong interpretability and clinical utility, serving as a reliable tool for guiding individualized surgical planning.

## 1 Introduction

According to the latest GLOBOCAN 2024 estimates jointly released by the International Agency for Research on Cancer (IARC) and the American Cancer Society (ACS), lung cancer remains the leading cause of cancer-related mortality worldwide (1). Advancements and the widespread adoption of CT scanning technology have significantly improved the detection and diagnosis of early-stage lung adenocarcinoma (2). Based on the 5th edition of the WHO Classification of Thoracic Tumours (2021), lung adenocarcinoma is classified into atypical adenomatous hyperplasia (AAH), adenocarcinoma *in situ* (AIS), minimally invasive adenocarcinoma (MIA), and invasive adenocarcinoma (IAC) (3). AAH, AIS, and MIA exhibit minimal invasiveness and a favorable prognosis, with a 5-year disease-free survival rate approaching 100% following surgery; thus, wedge resection or sublobar resection is typically recommended. Conversely, IAC is associated with a poorer prognosis and generally necessitates lobectomy with lymph node dissection (4, 5). Consequently, the accurate preoperative identification of tumor invasiveness is critical for guiding individualized surgical planning, as it directly impacts patient quality of life and long-term prognosis.

Currently, conventional imaging characteristics and pathological examination serve as the primary modalities for diagnosing lung cancer invasiveness. However, conventional imaging features are susceptible to subjective interpretation, while pathological assessment is inherently invasive and cannot facilitate non-invasive preoperative prediction, highlighting a critical clinical gap (6, 7). Radiomics has emerged as a prominent research frontier, enabling the extraction of high-throughput quantitative data from medical images (e.g., CT, MRI) to construct models that capture the underlying tumor microenvironment and pathophysiological information. This approach holds substantial value for differential diagnosis, classification, staging, and prognosis (8, 9). Nevertheless, existing radiomics models have notable limitations, as most studies focus exclusively on the intratumoral region. While our preliminary work demonstrated that combining conventional imaging with intratumoral radiomics effectively predicts invasive lung adenocarcinoma, the peritumoral region was not thoroughly investigated (10). Recently, emerging evidence suggests that peritumoral tissue, a crucial component of the tumor microenvironment, exhibits significant heterogeneity and actively influences tumorigenesis and progression (11). Despite this, the optimal peritumoral margin for predicting lung adenocarcinoma invasiveness remains undefined, and comprehensive models integrating conventional imaging with both intratumoral and peritumoral radiomics are still lacking (12). Furthermore, many traditional “black-box” models lack transparency, which hinders clinical trust and adoption (13). Therefore, this study aims to determine the optimal peritumoral distance to construct a combined conventional-intratumoral-peritumoral radiomics score model for predicting lung adenocarcinoma invasiveness. We developed a visualized nomogram and incorporate SHAP (SHapley Additive exPlanations) interpretability analysis to better inform clinical decision-making regarding surgical management.

## 2 Materials and methods

### 2.1 Study population

This retrospective study included 171 patients with pulmonary nodules who met the eligibility criteria at the Affiliated Hospital of Liaoning University of Traditional Chinese Medicine between January 2018 and December 2025. Inclusion criteria: (1) Pathological confirmation of AAH, AIS, MIA, or IAC following surgical resection; (2) Preoperative contrast-enhanced CT performed within 30 days of surgery; (3) Pulmonary nodules with a maximum diameter ≤ 30 mm on CT images; (4) No evidence of regional lymph node or distant metastasis on preoperative assessment; (5) Availability of complete clinicopathological and imaging data. Exclusion criteria: (1) Prior anti-tumor therapy (chemotherapy, radiotherapy, targeted therapy, or immunotherapy) directed at the nodule; (2) Primary tumors originating from extrathoracic organs or tissues; (3) Severe motion or metal artifacts on CT images that precluded effective nodule evaluation; (4)Patients with a history of or concurrent extrapulmonary malignancies elsewhere may be included. However, patients with a prior malignancy that has undergone curative surgical resection with no residual tumor post-surgery, excluding those who have received only radiotherapy, chemotherapy, targeted therapy, or immunotherapy, and for whom the interval from the last anticancer treatment to the date of this surgery is at least 5 years, with continuous disease-free survival of 5 years and no evidence of recurrence or metastasis, can be enrolled. Detailed clinical information, including sex, age, and smoking and alcohol consumption history, was recorded for all patients. This study extends Ref 10 (intratumoral-only radiomics) with 151/171 overlapping patients. The cohort was refined via stricter inclusion criteria, systematic re-screening, and one additional year of cases. Methodologically, it adds stepwise peritumoral exploration (0–8 mm), nested CV, Bootstrap.

Based on the latest WHO classification guidelines, cases were categorized into a pre-microinvasive lesion group (AAH, AIS, MIA) and an invasive lesion group (IAC) (3). Patients were randomly allocated into a training set (*n* = 120) and a test set (*n* = 51) at a 7:3 ratio. As a retrospective study, this research was approved by the Ethics Committee of the Affiliated Hospital of Liaoning University of Traditional Chinese Medicine [Approval No: Y2026014CS(KT)-014-01]. As this was a retrospective study, no prior sample size calculation was performed. Consecutive eligible patients from a single institution were included based on data availability. The final sample size is comparable to previous radiomics studies on lung adenocarcinoma invasiveness prediction (14, 15), and the nested cross-validation framework was employed to mitigate overfitting concerns associated with limited sample size.

### 2.2 CT acquisition

CT scans were acquired using either a Brilliance iCT or a Spectral CT7500 scanner. Patients were positioned supine with arms raised above the head and scanned in a head-first orientation, extending from the lung apex to the base. A non-ionic contrast agent (Ioversol, 350 mg/mL) was injected intravenously at a rate of 2.5–3.0 mL/s. Arterial phase and venous phase images were obtained 28 and 70 s after injection, respectively, during suspended respiration following a deep inspiration. Scan parameters were configured as follows: tube voltage was set at 120 kV for both scanners; tube current was 127 mA for the Brilliance iCT and auto-modulated for the Spectral CT7500; pitch was set at 0.758 and 0.8, respectively; collimation was 128 mm × 0.625 mm; rotation time was 0.5 s per rotation; and reconstruction thickness and interval were 0.9 mm for the Brilliance iCT and 1.0 mm for the Spectral CT7500.

### 2.3 Image analysis

Two chest radiologists, each with 5 years of experience, independently reviewed all images in a double-blind manner (blinded to the final pathological outcomes). Conventional imaging features assessed included the mean CT value, maximum diameter, density, margin characteristics (lobulation and spiculation), pleural traction, and vascular and bronchial changes. The mean CT value was measured by manually delineating a region of interest (ROI) on the section displaying the largest cross-sectional area of the nodule, as well as on the immediate adjacent sections above and below; the final value was calculated as the average of these three measurements. All CT value measurements were performed on arterial-phase contrast-enhanced images, as this phase was used for radiomics feature extraction to maintain consistency between conventional and radiomics features. Vascular changes were defined as enlargement, distortion, or convergence of vessels passing through the lesion. Bronchial changes were characterized by truncation, distortion, or enlargement of the bronchi traversing the lesion. Any disagreements between the two radiologists were resolved through consensus discussion.

### 2.4 Lesion segmentation and feature extraction

DICOM images of pulmonary nodules were exported from the PACS system and imported into the open-source software 3D Slicer (version 5.8.1). A chest radiologist with 5 years of experience manually segmented the intratumoral region of interest (ROI) slice-by-slice on arterial phase images (window width: 1400 HU; window level: −500 HU) in a blinded manner, carefully avoiding adjacent normal lung tissue. The software subsequently generated a volumetric ROI (VOI). To define the peritumoral regions, morphological dilation was applied to the intratumoral VOI with a 2 mm step size, generating four cumulative peritumoral margins (0–2 mm, 0–4 mm, 0–6 mm, and 0–8 mm) (each encompassing all inner rings). For nodules adjacent to the pleura or fissures, normal lung structures (airways, vessels, pleural tissue) within the dilated region were manually removed after automatic dilation, retaining only lung parenchyma for feature extraction. All segmentations were then reviewed and verified by a senior chest radiologist with extensive experience. Prior to feature extraction, images were resampled to an isotropic voxel spacing of 1 × 1 × 1 mm^3^ and discretized using a bin width of 25.0. Radiomic features were extracted using the Radiomics extension (version 5.8.1) within 3D Slicer (version 5.8.1), ensuring compliance with the Image Biomarker Standardization Initiative (IBSI) guidelines. These comprised 14 shape features, 18 first-order features, and 75 texture features, including those derived from the Gray Level Co-occurrence Matrix (GLCM), Gray Level Dependence Matrix (GLDM), Gray Level Run Length Matrix (GLRLM), Gray Level Size Zone Matrix (GLSZM), and Neighborhood Gray Tone Difference Matrix (NGTDM) (16). Wavelet and Laplacian of Gaussian (LoG) filtering were omitted to limit feature dimensionality and mitigate overfitting given the sample size (*n* = 171), preserving model interpretability and generalizability. To assess feature extraction reproducibility, 30 patients were randomly selected, and the initial radiologist repeated the segmentation after a 2-week interval. The intra-class correlation coefficient (ICC) was calculated to evaluate intra-observer reliability; Only features with an ICC > 0.75 were retained for subsequent modeling, as ICC values above this threshold are considered indicative of good reproducibility (17, 18). Finally, to eliminate differences in scale, all radiomics features were standardized using Z-score normalization (19).

### 2.5 Radiomics feature selection and model construction

Radiomics features extracted from the intratumoral VOI and the four peritumoral VOIs were separately screened using the Least Absolute Shrinkage and Selection Operator (LASSO) regression with 5-fold cross-validation. The *λ* value that minimized the cross-validation error was selected, and features with non-zero coefficients were retained as important predictors. Subsequently, binary logistic backward stepwise regression was performed on the selected features. The final radiomics score model was constructed by integrating these features into a logistic regression framework. The optimal peritumoral range was determined based on ROC and DCA results from the training set. To prevent information leakage, all feature selection and model fitting were performed within a nested resampling framework on the training set (*n* = 120): nested 5-fold cross-validation (outer 5 folds × inner 5 folds = 25 independent iterations of feature selection) and nested Bootstrap (1,000 iterations with out-of-bag evaluation, ∼44 cases per iteration). The test set was reserved exclusively for independent validation of the final model.

### 2.6 Development of clinical, radiomics, and combined models

Univariate analysis was first performed to screen clinical factors and conventional imaging features for potential associations with lung adenocarcinoma invasiveness (*P* < 0.05). Variables with statistical significance were then subjected to multivariate logistic regression analysis to identify independent risk factors and construct a conventional model.Subsequently, meaningful intratumoral and optimal peritumoral radiomics features were fed into a multivariable logistic algorithm to establish the intratumoral and peritumoral radiomics score model. Finally, significant features from both conventional imaging and radiomics analyses were fused to develop a combined conventional-intratumoral-optimal peritumoral radiomics score model, which was visualized as a nomogram. The predictive performance of all constructed models was assessed using ROC curve analysis and DCA. Internal validation of the final combined model was performed using the nested CV and nested Bootstrap procedures described in Section 2.5.

### 2.7 Statistical analysis

Statistical analyses were performed using SPSS version 27.0, the Zstats online platform (www.zstats.net), and R 4.5.2. Continuous variables with a normal distribution were presented as the mean ± standard deviation (SD) and compared using the independent-sample t-test. Continuous variables with a non-normal distribution were expressed as the median (M) with interquartile range (Q₁, Q₃) and analyzed using the Mann–Whitney U test. Categorical variables were described as frequencies and percentages [n (%)] and compared using either the Chi-square test or Fisher's exact test. A two-sided *P*-value of less than 0.05 was considered statistically significant. (Figure 1)

**Figure 1:**
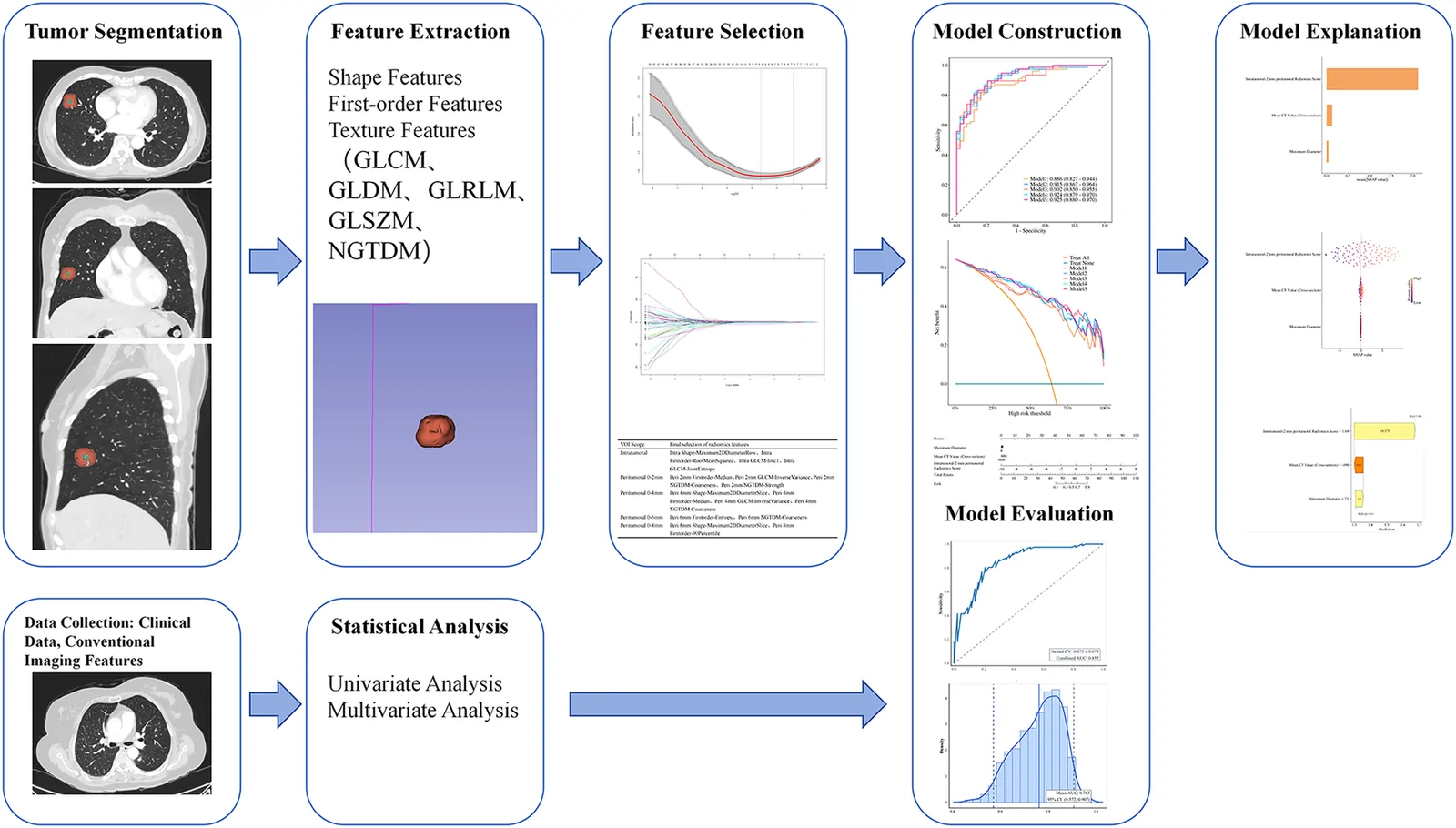
Workflow.

## 3 Results

### 3.1 Baseline characteristics and intergroup comparisons

In the training set, there were 43 pre-microinvasive lesions (AAH/AIS/MIA) and 77 invasive adenocarcinomas (IAC). In the independent test set, there were 17 pre-microinvasive and 34 IAC lesions. As summarized in Table 1, significant differences were observed in density, lobulation, pleural traction, maximum diameter, and mean CT value (cross-section) between the two groups in both the training and test sets (all *P* < 0.05). No statistically significant differences were found for the remaining clinical and conventional imaging characteristics (*P* > 0.05). Furthermore, multivariate logistic regression analysis of the training set identified maximum diameter and mean CT value (cross-section) as independent predictors for differentiating between pre-microinvasive and invasive lesions (both *P* < 0.05).

**Table 1:** Comparison of clinical data and conventional imaging features between the training set and the test set.

Variable	Training Set (*n* = 120)				Test Set (*n* = 51)			
	Preinvasive-MIA	IAC	t/Z/χ^2^	*P*	Preinvasive-MIA	IAC	t/Z/χ^2^	*P*
Gender			0.647	0.421			2.534	0.111
Male	20 (46.5%)	30 (39.0%)			5 (29.4%)	18 (52.9%)		
Female	23 (53.5%)	47 (61.0%)			12 (70.6%)	16 (47.1%)		
Age (year)	64.0 (55.0,71.0)	65.0 (58.0,71.0)	−0.644	0.520	59.1 ± 13.3	62.9 ± 8.8	1.217	0.229
Smoking history	14 (32.6%)	22 (28.6%)	0.209	0.648	1 (5.9%)	8 (23.5%)	1.366	0.242
Alcohol history	6 (14.0%)	11 (14.3%)	0.003	0.960	0 (0.0%)	7 (20.6%)	2.504	0.114
Lesion locations			8.527	0.070			2.845	0.643
Right upper lobe	11 (25.6%)	30 (39.0%)			7 (41.2%)	21 (61.8%)		
Right lower lobe	6 (14.0%)	10 (13.0%)			2 (11.8%)	3 (8.8%)		
Right middle lobe	5 (11.6%)	3 (3.9%)			3 (17.6%)	3 (8.8%)		
Left upper lobe	16 (37.2%)	16 (20.8%)			3 (17.6%)	5 (14.7%)		
Left lower lobe	5 (11.6%)	18 (23.4%)			2 (11.8%)	2 (5.9%)		
Density			6.427	0.011			6.007	0.014
Subsolid nodule	40 (93.0%)	57 (74.0%)			17 (100.0%)	22 (64.7%)		
Solid nodule	3 (7.0%)	20 (26.0%)			0 (0.0%)	12 (35.3%)		
Lobulation sign	3 (7.0%)	18 (23.4%)	5.140	0.023	0 (0.0%)	11 (32.4%)	5.230	0.022
Spiculation sign	2 (4.7%)	22 (28.6%)	9.867	0.002	0 (0.0%)	7 (20.6%)	2.504	0.114
Pleural traction	11 (25.6%)	51 (66.2%)	18.260	＜0.001	3 (17.6%)	16 (47.1%)	4.194	0.041
Vascular changes	2 (4.7%)	25 (32.5%)	12.243	＜0.001	1 (5.9%)	12 (35.3%)	3.730	0.053
Bronchial changes	0 (0.0%)	14 (18.2%)	8.851	0.003	0 (0.0%)	5 (14.7%)	1.358	0.244
Maximum Diameter (mm)	11.0 (8.0,16.0)	19.0 (14.0，24.5)	−5.377	＜0.001	13.0 (10.0,16.5)	17.5 (14.0,26.0)	−2.710	0.007
Mean CT Value (Cross-section)	−569.508 (−654.303，−501.436)	−356.245 (−493.573，−213.498)	−5.886	＜0.001	−587.515 ± 111.830	−330.512 ± 202.464	5.833	＜0.001

The t-test, Mann–Whitney U test, and Chi-square Test are employed.

### 3.2 Radiomics feature analysis

The ICC analysis of the 30 patients demonstrated that the 535 extracted radiomics features had ICC values ranging from 0.72 to 0.999. Two features from the 8 mm peritumoral region with an ICC < 0.75 were excluded, leaving 533 features for subsequent analysis. In the training set, the LASSO algorithm combined with binary logistic backward stepwise regression was applied to select features from the intratumoral and the four peritumoral ranges separately. This process retained 4, 4, 4, 2, and 2 optimal radiomics features for the intratumoral, 2 mm, 4 mm, 6 mm, and 8 mm peritumoral regions, respectively (Table 2, Figure 2, Supplementary Figures S1–S4). A radiomics score (Rad-score) was then constructed by weighting these selected features. This Rad-score was incorporated into the nomogram as an independent predictive variable. (Figure 7, Supplementary Data).

**Table 2:** List of five final VOI-selected radiomics features.

VOI Scope	Final selection of radiomics features
Intratumoral	Intra Shape-Maximum2DDiameterRow、Intra Firstorder-RootMeanSquared、Intra GLCM-Imc1、Intra GLCM-JointEntropy
Peritumoral 0–2mm	Peri 2 mm Firstorder-Median、Peri 2 mm GLCM-InverseVariance、Peri 2 mm NGTDM-Coarseness、Peri 2 mm NGTDM-Strength
Peritumoral 0–4mm	Peri 4 mm Shape-Maximum2DDiameterSlice、Peri 4 mm Firstorder-Median、Peri 4 mm GLCM-InverseVariance、Peri 4 mm NGTDM-Coarseness
Peritumoral 0–6mm	Peri 6 mm Firstorder-Entropy、Peri 6 mm NGTDM-Coarseness
Peritumoral 0–8mm	Peri 8 mm Shape-Maximum2DDiameterSlice、Peri 8 mm Firstorder-90Percentile

**Figure 2:**
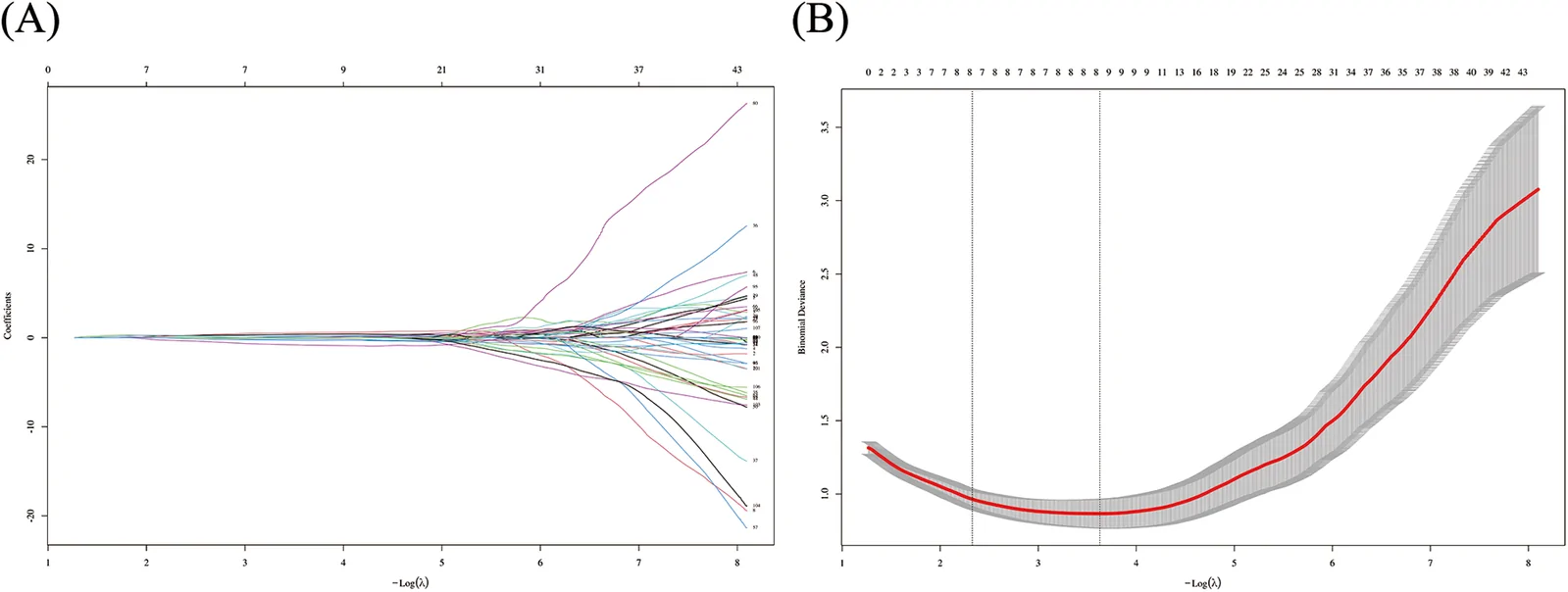
Intratumoral VOI lasso regression results: **(A)**: path plot of lasso regression coefficients; **(B)**: lasso regression performed using five-fold cross-validation to identify the optimal log(*λ*) value.

### 3.3 Model evaluation

#### 3.3.1 Comparison of peritumoral radiomics score models

In the training set, the 2 mm peritumoral radiomics score model achieved an AUC of 0.902 (95% CI: 0.850–0.955), outperforming the 4 mm (AUC = 0.888; 95% CI: 0.832–0.944), 6 mm (AUC = 0.885; 95% CI: 0.827–0.944), and 8 mm (AUC = 0.870; 95% CI: 0.809–0.931) models. Compared with the other three models, the 2 mm model yielded NRIs of 0.3727 (*P* = 0.04493), 0.5805 (*P* = 0.00148), and 0.3467 (*P* = 0.06237), respectively. Specifically, the IDI of the 2 mm model relative to the 8 mm model was 0.0734 (*P* = 0.00915). Pairwise DeLong tests were performed to compare the diagnostic performance of the 2-mm peritumoral model against the 4-mm, 6-mm, and 8-mm models. In the training set, no statistically significant differences were observed (all *P* > 0.05).These results further validated the trend that the 2 mm peritumoral margin exhibited optimal performance in the present cohort. In the independent test set, the 2 mm model achieved the highest AUC (0.881; 95% CI: 0.787–0.974) among the four peritumoral margins. Pairwise DeLong tests identified a significant performance difference only between the 2 mm and 6 mm models (*P* < 0.05); other comparisons were non-significant (all *P* > 0.05). Furthermore, compared with the 4 mm and 8 mm models, the 2 mm model in the test set yielded NRIs of 0.6471 (*P* = 0.02173) and 0.5294 (*P* = 0.0637), and IDIs of 0.0929 (*P* = 0.02146) and 0.1224 (*P* = 0.0029), respectively. The overlapping 95% CIs observed among several models reflect the limited statistical precision afforded by the test-set sample size (*n* = 51)(Figures 3, 4, 5, Supplementary Tables 1, 2).

**Figure 3:**
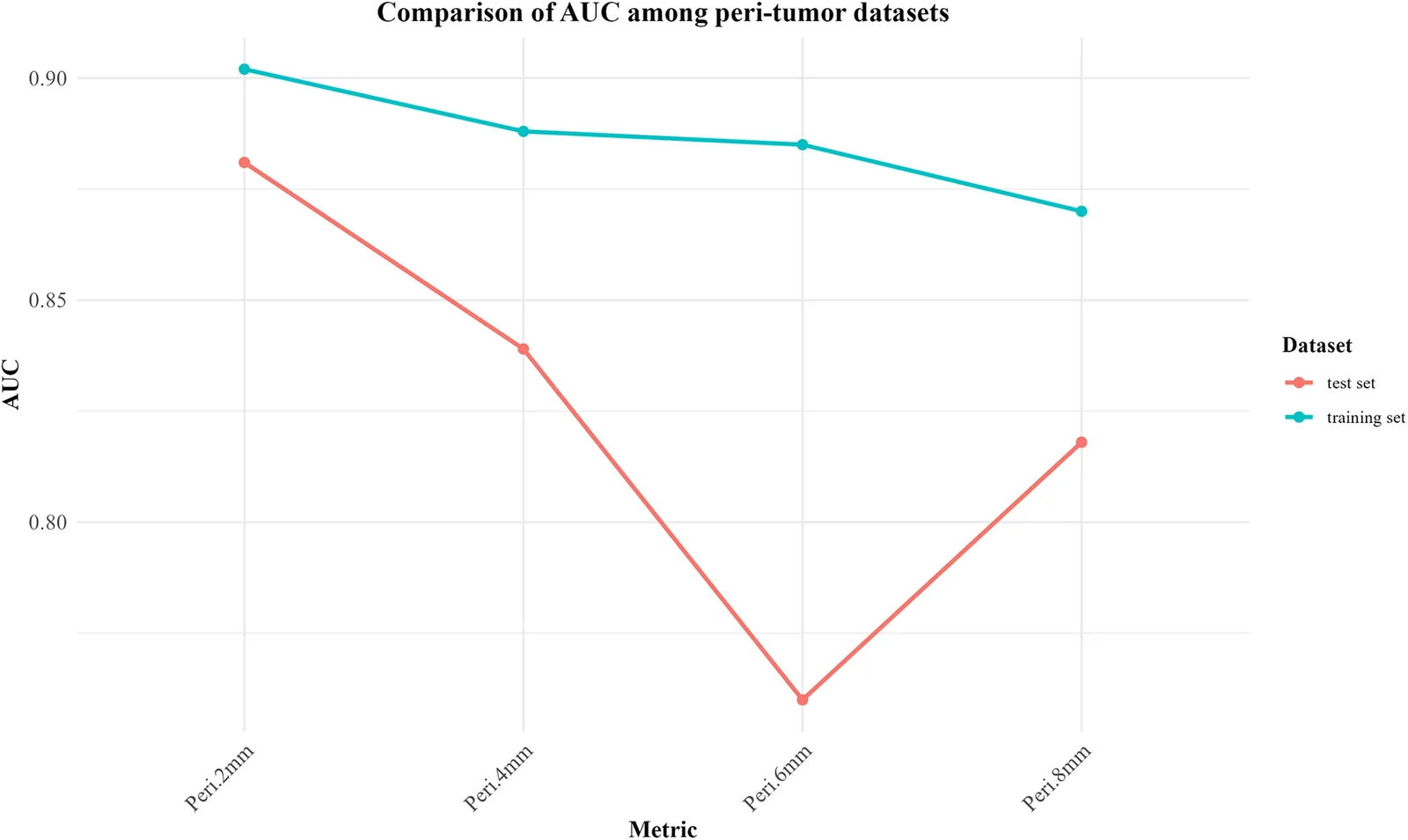
Comparison of four peritumoral radiomics score models.

**Figure 4:**
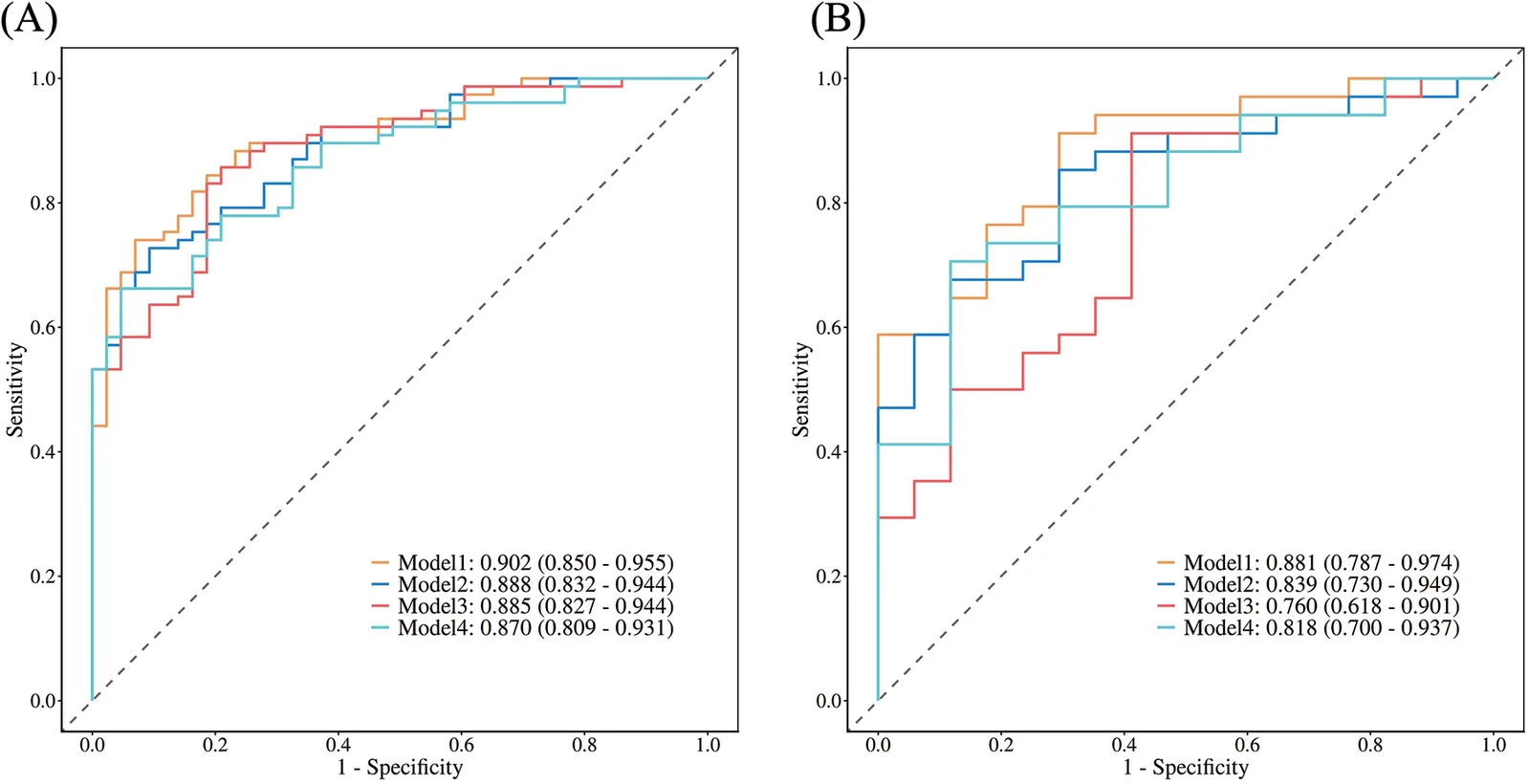
ROC curves of the four peritumoral radiomics score models on the training and test sets. **(A)**: ROC curves of the four models on the training set; **(B)**: ROC curves of the four models on the test set.

**Figure 5:**
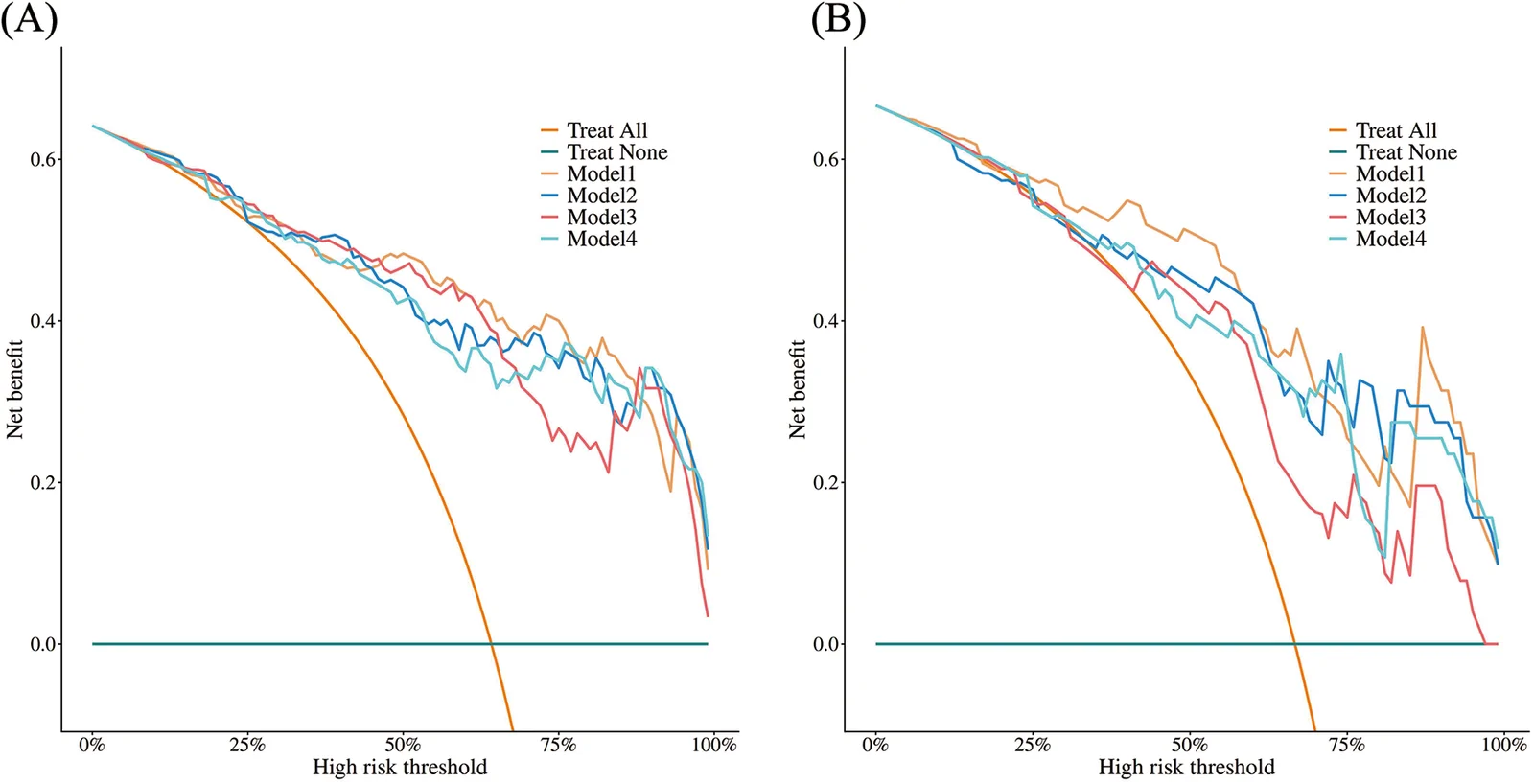
DCA curves of the four peritumoral radiomics score models on the training and test sets. **(A)**: DCA curves of the four models on the training set; **(B)**: DCA curves of the four models on the test set.

#### 3.3.2 Performance comparison of conventional and radiomics models

Based on the optimal 2 mm peritumoral margin identified in the training set, a combined conventional-intratumoral-2 mm peritumoral radiomics score model was developed. In the training set, this combined model achieved an AUC of 0.925 (95% CI: 0.880–0.970), outperforming the conventional model (AUC = 0.886; 95% CI: 0.827–0.944), the intratumoral radiomics score model (AUC = 0.915; 95% CI: 0.867–0.964), the 2 mm peritumoral radiomics score model (AUC = 0.902; 95% CI: 0.850–0.955), and the intratumoral-2 mm peritumoral radiomics score model (AUC = 0.924; 95% CI: 0.879–0.944) (Table 3). DeLong tests confirmed that the combined model was significantly superior to the conventional model (*P* = 0.03311), with an NRI of 0.5545 (*P* = 0.00247) and an IDI of 0.1061 (*P* = 0.00033). Compared to the 2 mm peritumoral model, the combined model yielded an NRI of 0.4561 (*P* = 0.01398) and an IDI of 0.0654 (*P* = 0.01532). In the independent test set, the combined model maintained the highest predictive performance, with an AUC of 0.888 (95% CI: 0.796–0.979) (Table 4, Figure 6). In the test set, pairwise DeLong tests between the combined model and conventional model, as well as between the combined model and single-modality radiomics models, yielded non-significant results (all *P* > 0.05), consistent with the limited statistical power of the test cohort (*n* = 51) for comparative testing. The nested cross-validation results from the training set therefore provide the primary evidence for incremental value assessment. To facilitate clinical application, a visualized nomogram was constructed based on the combined model (Figure 7). The calibration curves indicated excellent agreement between the predicted probabilities and actual observations (Hosmer-Lemeshow test *P* = 0.952 and 0.733 for the training and test sets, respectively; Figure 8). Furthermore, decision curve analysis (DCA) demonstrated that the combined model provided a favorable net clinical benefit across a wide range of threshold probabilities (10%–90%), supporting its clinical utility in preoperative decision-making (Figure 9). Nested 5-fold cross-validation yielded a mean AUC of 0.873 ± 0.079, and nested Bootstrap (1,000 iterations) yielded a mean AUC of 0.763 (95% CI: 0.572–0.907), confirming model robustness without information leakage (Figure 10). The clinical utility of the nomogram across the two patient groups is illustrated in Figures 11, 12.

**Table 3:** Predictive performance of the five models on the training set.

Model	AUC (95%CI)	Accuracy (95%CI)	Sensitivity (95%CI)	Specificity (95%CI)	PPV (95%CI)	NPV (95%CI)	F1 score	Brier score	Cut-off
Model1	0.886 (0.827–0.944)	0.833 (0.754–0.895)	0.857 (0.779–0.935)	0.791 (0.669–0.912)	0.880 (0.806–0.954)	0.756 (0.630–0.881)	0.868	0.132	0.5050434
Model2	0.915 (0.867–0.964)	0.833 (0.754–0.895)	0.818 (0.732–0.904)	0.860 (0.757–0.964)	0.913 (0.847–0.980)	0.725 (0.603–0.848)	0.863	0.113	0.6447314
Model3	0.902 (0.850–0.955)	0.808 (0.726–0.874)	0.740 (0.642–0.838)	0.930 (0.854–1.000)	0.950 (0.895–1.000)	0.667 (0.547–0.786)	0.832	0.124	0.7376292
Model4	0.924 (0.879–0.970)	0.842 (0.764–0.902)	0.818 (0.732–0.904)	0.884 (0.788–0.980)	0.926 (0.864–0.989)	0.731 (0.610–0.851)	0.869	0.108	0.6708207
Model5	0.925 (0.880–0.970)	0.842 (0.764–0.902)	0.831 (0.747–0.915)	0.860 (0.757–0.964)	0.914 (0.849–0.980)	0.740 (0.618–0.862)	0.871	0.108	0.6509728

Model1: Conventional model, Model2: Intratumoral radiomics score model, Model3: 2 mm peritumoral radiomics score model, Model4: Intratumoral-2 mm peritumoral radiomics score model, Model5: Conventional-intratumoral-2 mm peritumoral radiomics score model. AUC: Area under the curve; CI: Confidence interval; PPV: Positive predictive value; NPV: Negative predictive value.

**Table 4:** Predictive performance of the five models on the test set.

Model	AUC (95%CI)	Accuracy (95%CI)	Sensitivity (95%CI)	Specificity (95%CI)	PPV (95%CI)	NPV (95%CI)	F1 score	Brier score	Cut-off
Model1	0.879 (0.785–0.973)	0.824 (0.691–0.916)	0.824 (0.695–0.952)	0.824 (0.642–1.000)	0.903 (0.799–1.000)	0.700 (0.499–0.901)	0.862	0.134	0.6481308
Model2	0.881 (0.776–0.985)	0.843 (0.714–0.930)	0.794 (0.658–0.930)	0.941 (0.829–1.000)	0.964 (0.896–1.000)	0.696 (0.508–0.884)	0.871	0.125	0.7668496
Model3	0.881 (0.787–0.974)	0.843 (0.714–0.930)	0.912 (0.816–1.000)	0.706 (0.489–0.922)	0.861 (0.748–0.974)	0.800 (0.598–1.000)	0.886	0.133	0.5171168
Model4	0.886 (0.794–0.978)	0.843 (0.714–0.930)	0.853 (0.734–0.972)	0.824 (0.642–1.000)	0.906 (0.805–1.000)	0.737 (0.539–0.935)	0.879	0.127	0.5843251
Model5	0.888 (0.796–0.979)	0.804 (0.669–0.902)	0.735 (0.587–0.884)	0.941 (0.829–1.000)	0.962 (0.888–1.000)	0.640 (0.452–0.828)	0.833	0.128	0.8224447

Model1: Conventional model, Model2: Intratumoral radiomics score model, Model3: 2 mm peritumoral radiomics score model, Model4: Intratumoral-2 mm peritumoral radiomics score model, Model5: Conventional-intratumoral-2 mm peritumoral radiomics score model. AUC: Area under the curve; CI: Confidence interval; PPV: Positive predictive value; NPV: Negative predictive value.

**Figure 6:**
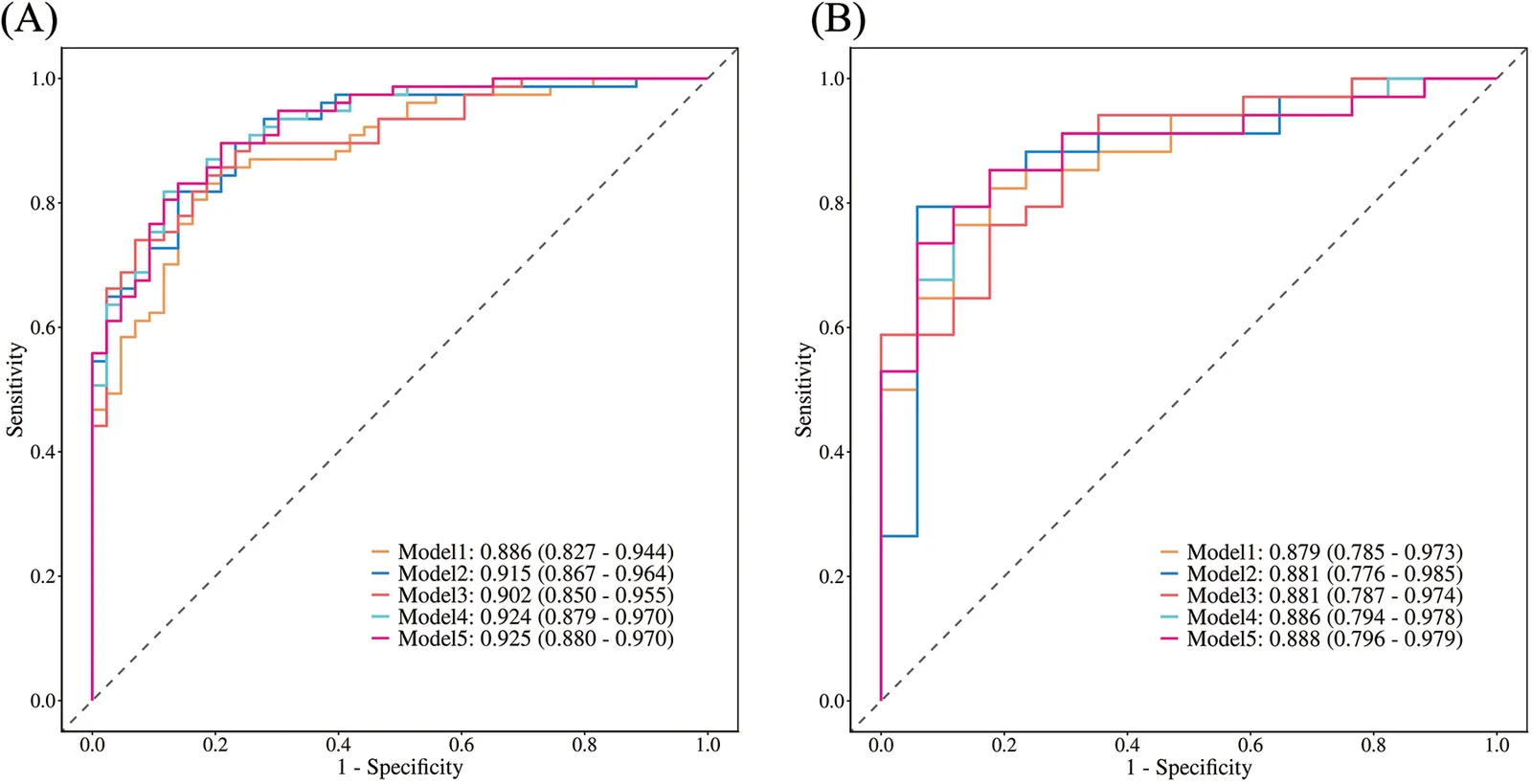
ROC curves of the five models on the training and test sets. **(A)**: ROC curves for the five models on the training set; **(B)**: ROC curves for the five models on the test set.

**Figure 7:**
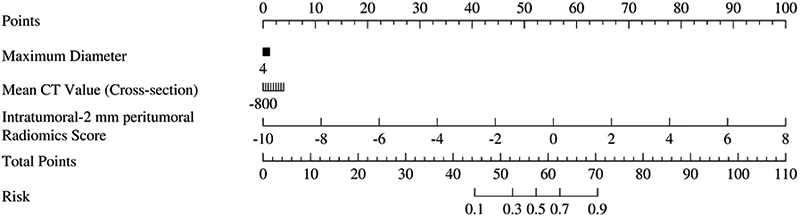
Conventional-intratumoral-2 mm peritumoral radiomics score model nomogram.

**Figure 8:**
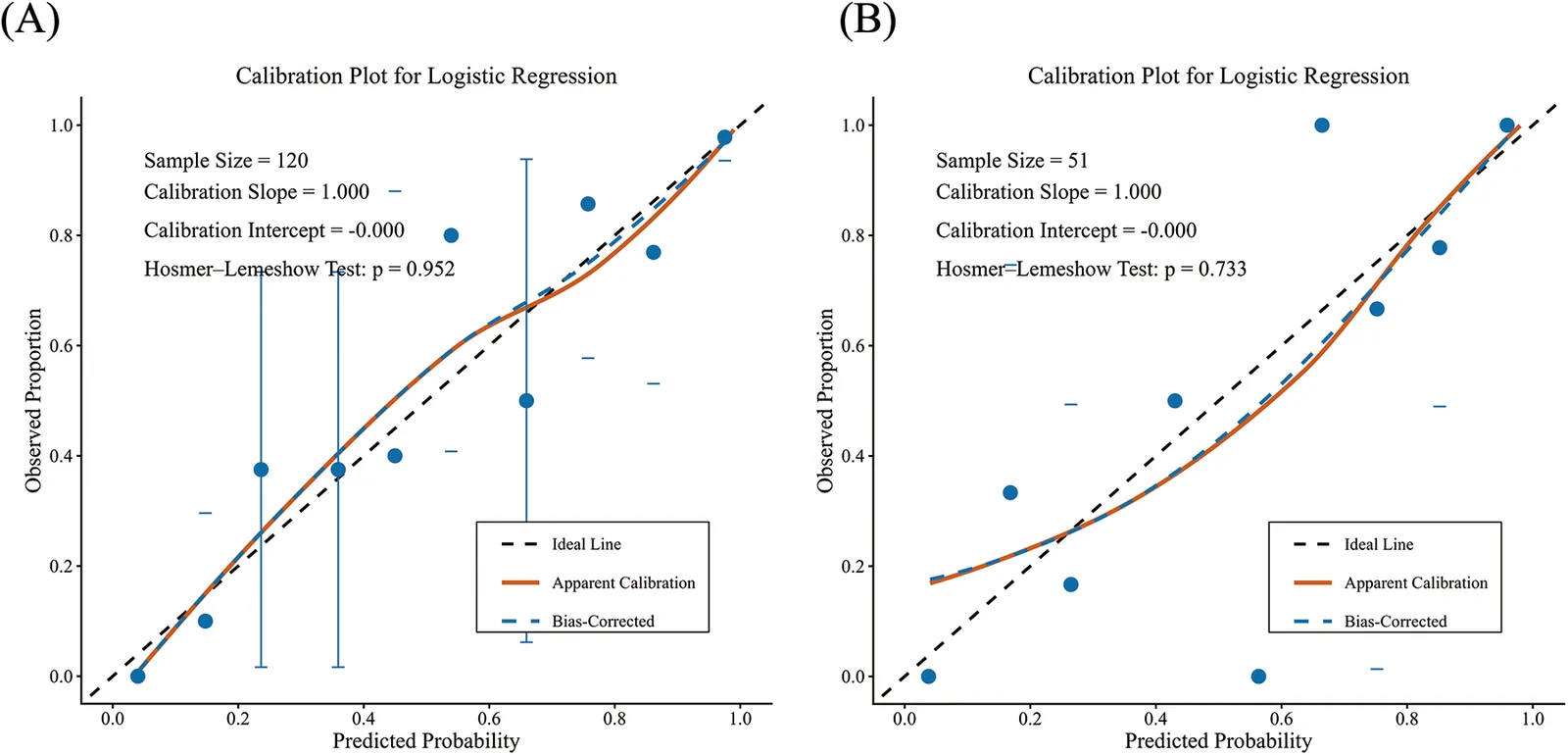
Calibration curves of the conventional-intratumoral-2 mm peritumoral radiomics score model on the training and test sets. **(A)**: Calibration curve of the conventional-intratumoral-2 mm peritumoral radiomics score model on the training set; **(B)**: Calibration curve of the conventional-intratumoral-2 mm peritumoral radiomics score model on the test set.

**Figure 9:**
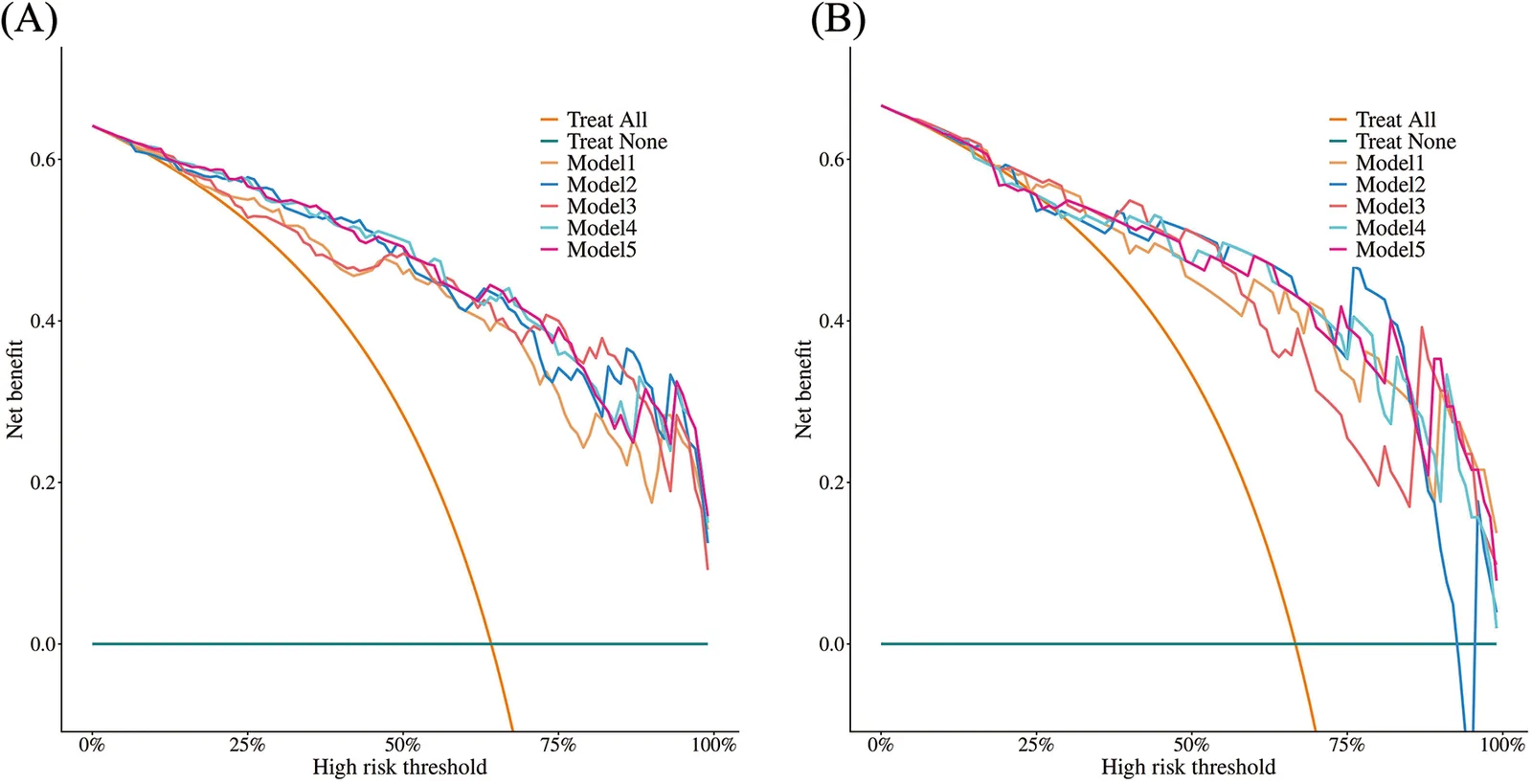
DCA curves of the five models on the training and test sets. **(A)**:DCA curves for the five models on the training set; **(B)**: DCA curves for the five models on the test set.

**Figure 10:**
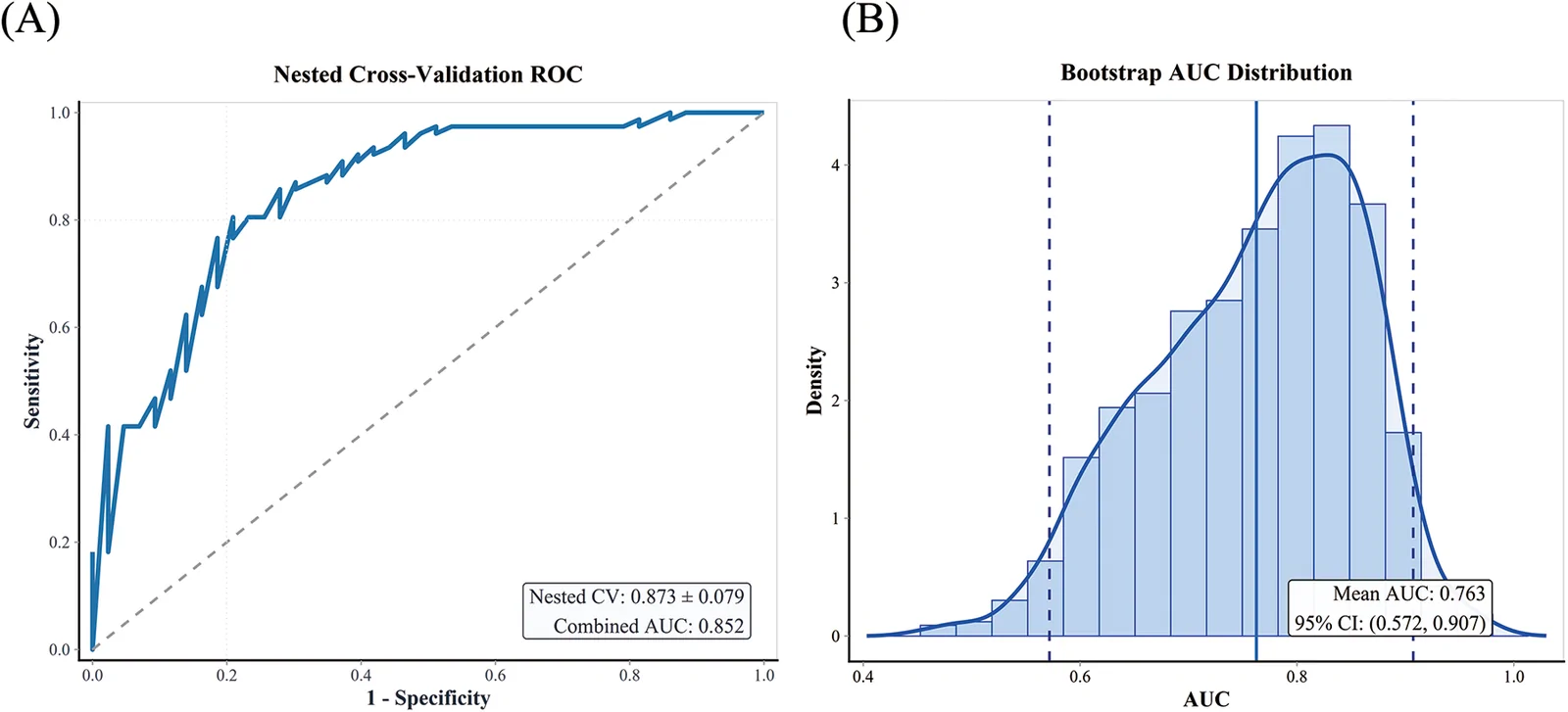
Model performance evaluation based on nested cross-validation and Bootstrap resampling. **(A)**:Nested cross-validation ROC curve; **(B)**: Bootstrap AUC distribution.

**Figure 11:**
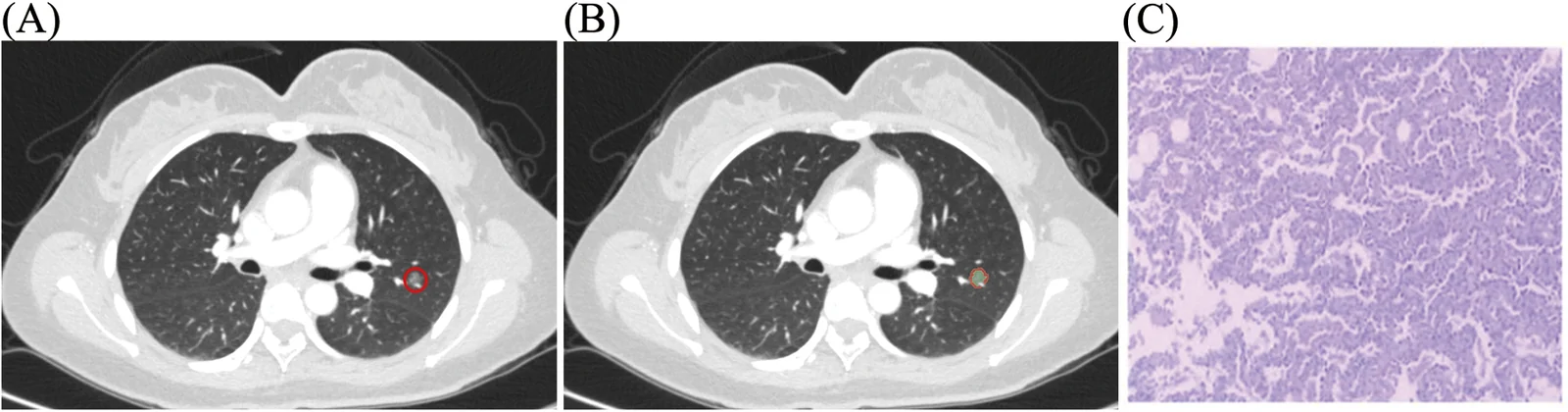
Case presentation of the preinvasive-MIA lesion group. **(A)**: The red circle delineates the approximate range of the ROI for the lesion; **(B)**: Green area: intratumoral region; red area: 0-2 mm peritumoral zone; **(C)**: Pathological diagnosis: (the left upper lobe of lung) minimally invasive adenocarcinoma. The maximum diameter of the nodule is 8 mm, and the mean CT value (cross-section) = −499.987 HU. The intratumoral-2 mm peritumoral radiomics score = −2.223662218, the total nomogram score = 45, corresponding to an invasive risk of approximately 10%.

**Figure 12:**
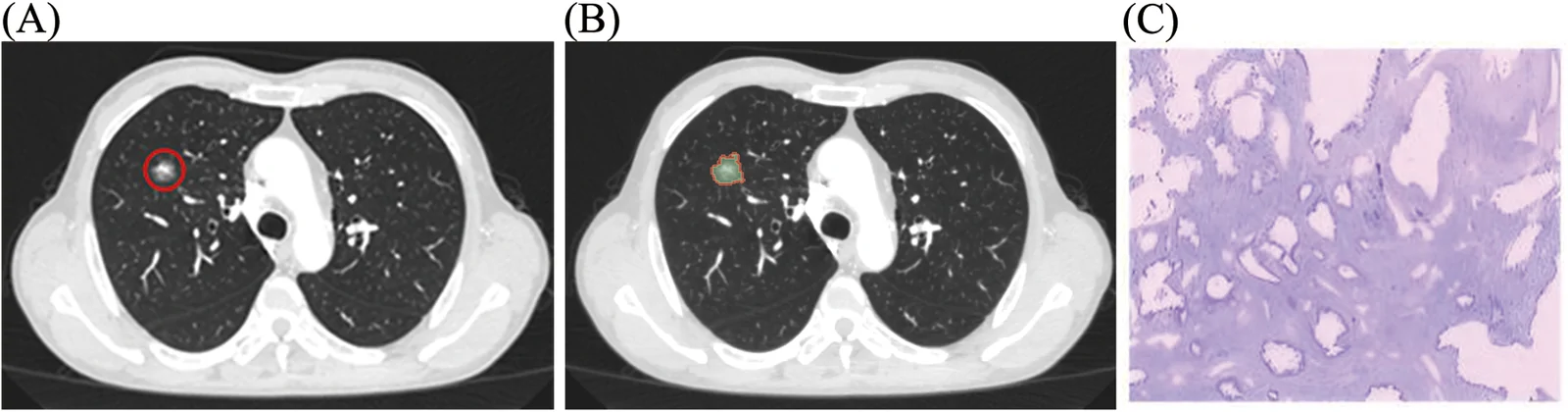
Case presentation of the invasive lesion group. **(A)**: The red circle delineates the approximate range of the ROI for the lesion; **(B)**: Green area: intratumoral region; red area: 0-2 mm peritumoral zone; **(C)**: Pathological diagnosis: (the right upper lobe of lung) invasive adenocarcinoma. The maximum diameter of the nodule is 16 mm, and the mean CT value (cross-section) = −460.226 HU. The intratumoral-2 mm peritumoral radiomics score = 1.51594059, the total nomogram score = 65, corresponding to an invasive risk of approximately 75%.

### 3.4 Model interpretation

The model feature importance analysis revealed that the three most significant predictors, in descending order, were the intratumoral-2 mm peritumoral radiomics score, mean CT value (cross-section), and maximum diameter. The SHAP summary swarm plot demonstrated that these three features acted as positive contributors to the model's output (Figure 13). As illustrated in the waterfall plot for a representative case (Figure 13), the base value (expected model output, E(f(x))) was 1.31. The intratumoral-2 mm peritumoral radiomics score (value = 1.69; SHAP = + 0.375) and maximum diameter (value = 23; SHAP = + 0.0487) pushed the prediction higher. Conversely, the mean CT value (cross-section = −490; SHAP = −0.0565) was the primary factor pulling down the predicted value. The combined effect of these features resulted in a final predicted value (f(x)) of 1.68.

**Figure 13:**
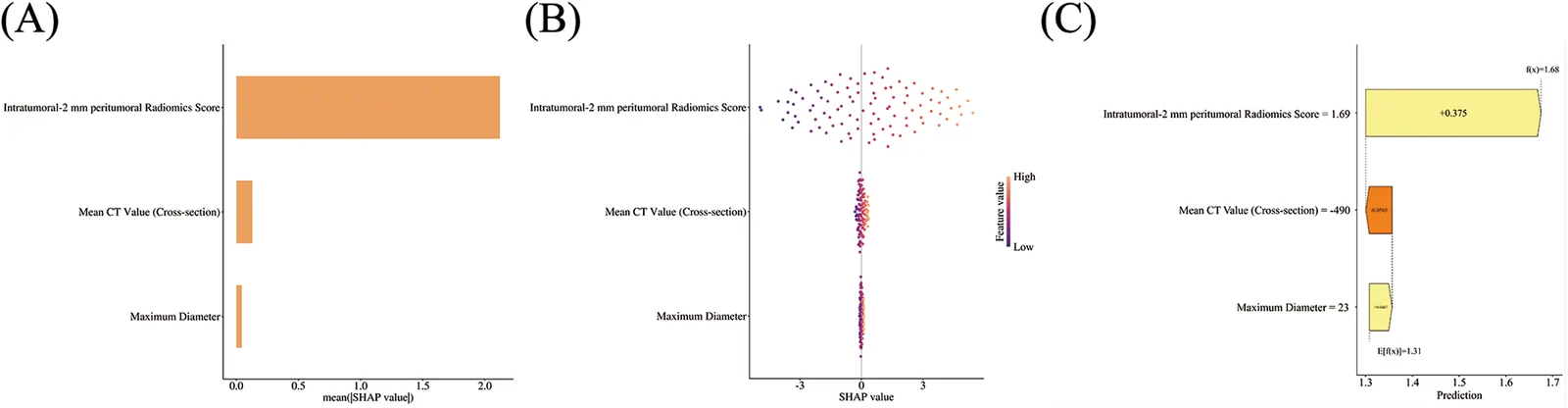
SHAP analysis of the conventional-intratumoral-2 mm peritumoral radiomics score model. **(A)**: SHAP plot of variable importance in the conventional-intratumoral-2 mm peritumoral radiomics score model; **(B)**: SHAP swarm plot of model features in the conventional-intratumoral-2 mm peritumoral radiomics score model; **(C)**: Waterfall Plot of conventional-intratumoral-2 mm peritumoral radiomics score model.

## 4 Discussion

In this study, we identified a 2 mm peritumoral margin as the optimal range for discriminating the invasive degree of pulmonary nodules in the present cohort. The nomogram that integrates conventional imaging features with intratumoral and 2 mm peritumoral radiomics features demonstrated good discriminatory ability, thereby providing a reliable preliminary tool for clinical decision-making.

Prior to the widespread adoption of radiomics, the diagnosis of lung adenocarcinoma invasiveness primarily relied on conventional CT imaging features. Features such as pleural traction, lobulation, spiculation, maximum diameter, and CT attenuation values have been shown to increase the likelihood of invasive lung adenocarcinoma (20). In the current study, multivariate analysis confirmed that maximum diameter and mean CT value (cross-section) are independent risk factors for determining nodule invasiveness. A larger lesion diameter correlates with a higher probability of malignancy (21). Furthermore, an elevated mean CT value reflects dense cellular proliferation and a greater proportion of solid components, which aligns with the highly invasive nature of pulmonary nodules. This finding is consistent with multiple previous investigations (22, 23).

In contrast, conventional features are susceptible to subjective interpretation (6, 7), motivating the use of radiomics for more objective, high-throughput quantification of tumor heterogeneity. Radiomics offers superior predictive value over conventional imaging for lung adenocarcinoma invasiveness (24–26). However, most existing radiomics studies have focused exclusively on the intratumoral region, leaving the peritumoral area relatively underexplored. While intratumoral radiomics reflects internal microscopic characteristics, the peritumoral tissue adjacent to the primary tumor—a crucial component of the tumor microenvironment (TME)—also actively influences tumor progression and outcome (27). The choice of arterial-phase imaging in this study was deliberate. Arterial phase contrast-enhanced CT captures heterogeneity in lesion perfusion during the first pass of iodinated contrast within tumor neovasculature. The degree of enhancement correlates positively with tumor microvessel density (MVD) (28, 29). It has been established that peritumoral MVD serves as an independent prognostic factor for lung adenocarcinoma (30). The invasive pathological features of lung adenocarcinoma involve TME alterations—such as increased MVD, stromal remodeling, and macrophage infiltration in both intratumoral and peritumoral regions—which play pivotal roles in tumor evolution (31, 32). While most prior studies focused exclusively on the intratumoral region, emerging evidence supports combined intratumoral-peritumoral models (33, 34). Nevertheless, the underlying mechanisms and optimal peritumoral margins for distinguishing invasiveness remain poorly defined in prior literature, hindering a clear understanding of peritumoral contributions to invasion risk. Therefore, drawing on the pathological transition zone (ZOT) width reported by Zhang et al., we employed a stepwise expansion (2, 4, 6, and 8 mm) to define peritumoral regions. This approach allows for the detection of radiomic signals across the direct infiltration interface (approx. 2 mm ring), the invasive front associated with high-grade histology (4 mm ring), and the broader peritumoral microenvironment (6–8 mm ring) (35). Our results revealed a gradual decline in model performance as the peritumoral range expanded. Signal dilution is an expected, inherent effect of cumulative peritumoral margins. However, this expected dilution mirrors the biological reality that invasive adenocarcinoma predominantly affects the immediate peritumoral zone (≤2 mm), beyond which stromal alterations become sparse (36, 37). Cumulative margins were adopted to preserve this zone. Future work using distance-weighted features may refine this approach. Pairwise DeLong tests supported the selection of the 2-mm margin: no significant differences among the four models in the training set (all *p* > 0.05) indicated a “trend toward optimal,” while the 2-mm model significantly outperformed the 6-mm model in the test set (*p* < 0.05), consistent with overfitting of wider margins. Compared with prior studies using fixed 5-mm margins (37, 39), our stepwise 0–8 mm evaluation more precisely captured peritumoral microenvironmental information, and the intratumoral-plus-2-mm model improved performance from 0.915 to 0.924, confirming the incremental value of peritumoral radiomics. Compared to our prior intratumoral-only study (10) (AUC = 0.938), the present models were developed on distinct but partially overlapping cohorts; rather than pursuing marginal AUC gains over a previous single-center cohort, this study prioritized systematic peritumoral evaluation, methodological rigor (nested CV/Bootstrap), and clinical interpretability. In this study, while the peritumoral model demonstrated predictive potential, intratumoral radiomics alone remained slightly superior. Integrating both features produced a synergistic effect, indicating that intratumoral characteristics are still the core imaging biomarker for assessing invasiveness, whereas peritumoral information provides a critical complementary dimension to this evaluation system.

Regarding the combined model, its test-set AUC (0.888) was nearly identical to that of the intratumoral (0.881) and conventional (0.879) models, indicating limited incremental value in terms of pure discriminative performance. Notably, its sensitivity (0.735) was lower than that of the conventional (0.824) and intratumoral (0.794) models, reflecting a sensitivity–specificity trade-off. Given the clinical goal of avoiding missed invasive adenocarcinoma, this sensitivity reduction may lead to underestimation of invasion risk; conversely, the higher specificity may benefit scenarios prioritizing avoidance of unnecessary sublobar resection. Nevertheless, the objective of constructing a clinical-radiomics combined model extends beyond merely maximizing AUC; rather, it aims to enhance clinical interpretability and translational potential. In this study, the combined model incorporated conventional imaging features—such as mean CT value and maximum diameter—that are highly familiar to clinicians. These features reflect macroscopic morphology visible to the naked eye, providing an intuitive clinical entry point for understanding model decisions. SHAP analysis further underscored the significant contribution of these conventional features, which, alongside radiomics features, collectively drive the predictive outcome (38). While intratumoral and peritumoral radiomics reveal microscopic heterogeneity (e.g., pixel-level distributions of tumor cell density, interstitial collagenization, and necrosis) through high-throughput extraction of texture, shape, and first-order statistical features, radiomics models inherently possess a “black-box” nature. The integration of conventional features ensures that even when radiomics scores are similar, clinicians can rapidly assess the model's reliability and directional tendency using familiar parameters. Consequently, this combined approach holds substantially greater clinical adoption value compared to an entirely opaque pure-radiomics model. Furthermore, the tumor core radiomics captures dense cellular and structural information that directly reflects lung adenocarcinoma invasiveness, whereas the peritumoral region—representing the invasive front and host stromal response—offers novel insights not fully captured by the intratumoral phenotype alone. These three information sources provide complementary strengths, comprehensively reflecting the tumor's growth dynamics and enabling a characterization of pulmonary nodules that spans from macroscopic presentation to local biological behavior.

The training-set AUC (0.925) represents an optimistic estimate expectedly higher than the test (0.888) and nested CV (0.873 ± 0.079) results. This discrepancy reflects expected model optimism rather than severe overfitting, as evidenced by the close convergence between nested CV and test performance. The nested CV mean AUC (0.873 ± 0.079) closely approximated the test result, while nested Bootstrap (1,000 iterations, ∼44 OOB cases) yielded 0.763 (95% CI: 0.572–0.907); the wide interval reflects limited OOB sample size but fully encompasses the test result, supporting robustness.

However, several limitations should be acknowledged. First, the single-center, retrospective design and relatively small sample size may have introduced selection bias. While nested cross-validation provided an unbiased internal performance estimate (mean AUC 0.873 ± 0.079), the nested Bootstrap analysis yielded a wider 95% CI (0.572–0.907) due to the limited out-of-bag sample size (∼44 cases per iteration). Therefore, the generalizability of the model requires independent evaluation through multi-center external validation. Second, mean CT values were obtained from arterial-phase contrast-enhanced images rather than non-contrast CT. Contrast-induced HU elevation may limit transportability, particularly since most included nodules were subsolid with minimal enhancement. The model is best suited for institutions routinely using contrast-enhanced CT; future work should compare imaging phases. Third, the subjectivity inherent in manual delineation and variability arising from multi-vendor scanners may interfere with feature extraction. Feature stability was assessed via intra-observer ICC in 30 randomly selected patients. However, inter-observer variability was not evaluated in this study, which may affect generalizability. Future studies should expand the sample size, incorporate multi-observer and automated segmentation tools, and optimize standardization pipelines to minimize this bias and enhance model generalizability. Fourth, this study was limited to a radiomics-level analysis. Subsequent research should integrate multi-dimensional data—such as pathomics and immunomics—and incorporate long-term follow-up information to construct a more clinically valuable comprehensive prediction model.

## 5 Conclusion

In conclusion, this study established a 2 mm peritumoral margin as the optimal radiomics research range for predicting lung adenocarcinoma invasiveness. By integrating the macroscopic morphological characteristics of conventional imaging with the microscopic heterogeneity of the intratumoral and peritumoral microenvironment, we constructed a combined conventional-intratumoral-2 mm peritumoral radiomics score model. Coupled with SHAP interpretability analysis and a visualized nomogram, this study provides clinicians with a novel preliminary reference tool that combines accuracy and transparency, thereby facilitating the accurate assessment of tumor invasiveness and the formulation of individualized surgical plans.

## Data Availability

The original contributions presented in the study are included in the article/Supplementary Material, further inquiries can be directed to the corresponding author.
