# Supplementary material for: A nomogram model for preoperative evaluation of invasive degree in lung adenocarcinoma: integrating intratumoral and 2 mm peritumoral radiomics scores with conventional features
Source: Front Med (Lausanne). 2026 Oct 28;13:1984781. doi: 10.3389/fmed.2026.1984781 (PMC13751333; doi:10.3389/fmed.2026.1984781)
Supplement: Supplementary file 1 [file Datasheet1.zip › Supplementary Material/Supplementary_Material.docx]

Supplementary Material

# 1 Supplementary Data

The formula for the radiomics score is as follows:

$$\mathrm{Intratumoral} Rad-score=1.3+1.294\times Intra Shape-Maximum2DDiameterRow-0.969\times Intra Firstorder-RootMeanSquared+0.631\times Intra GLCM-Imc1+0.968\times Intra GLCM-JointEntropy$$

$$\mathrm{Peritumoral}0-2\mathrm{mm}Rad-score=1.114+1.078\times\mathrm{Peri} 2mm \mathrm{Firstorder}-\mathrm{Median}-0.941\times\mathrm{Peri} 2mm \mathrm{GLCM}-\mathrm{InverseVariance}-1.334\times\mathrm{Peri} 2mm \mathrm{NGTDM}-\mathrm{Coarseness}-0.810\times\mathrm{Peri} 2mm \mathrm{NGTDM}-\mathrm{Strength}$$

$$Peritumoral 0-4mm Rad-score=1.296+1.046\times Peri 4mm Shape-Maximum2DDiameterSlice+0.801\times Peri 4mm Firstorder-Median-0.805\times Peri 4mm GLCM-InverseVariance-0.841\times Peri 4mm NGTDM-Coarseness$$

$$Peritumoral 0-6mm Rad-score=0.997+1.428\times Peri 6mm Firstorder-Entropy-1.295\times Peri 6mm NGTDM-Coarseness$$

$$Peritumoral 0-8mm Rad-score=1.5+2.22\times Peri 8mm Shape-Maximum2DDiameterSlice+2.01\times Peri 8mm Firstorder-90Percentile$$

$$\mathrm{Intratumoral}-Peritumoral 0-2mm Rad-score=1.29+0.806\times Intra Shape-Maximum2DDiameterRow-0.925\times Intra Firstorder-RootMeanSquared+0.344\times Intra GLCM-Imc1+0.827\times Intra GLCM-JointEntropy+0.698\times Peri 2mm Firstorder-Median+0.114\times Peri 2mm GLCM-InverseVariance-0.476\times Peri 2mm NGTDM-Coarseness-0.524\times Peri 2mm NGTDM-Strength$$

# 2 Supplementary Tables

**Supplementary Table 1.** Prediction performance of Four Peritumoral Radiomics Models in the training set.

| Model | AUC (95%CI) | Accuracy (95%CI) | Sensitivity (95%CI) | Specificity (95%CI) | PPV (95%CI) | NPV (95%CI) | F1 score | Brier score | Cut-off |
| --- | --- | --- | --- | --- | --- | --- | --- | --- | --- |
|  |  |  |  |  |  |  |  |  |  |
| Model1 | 0.902 (0.850-0.955) | 0.808 (0.726-0.874) | 0.740 (0.642 - 0.838) | 0.930 (0.854 - 1.000) | 0.950 (0.895 - 1.000) | 0.667 (0.547 - 0.786) | 0.832 | 0.124 | 0.7376008 |
| Model2 | 0.888 (0.832-0.944) | 0.792 (0.708-0.860) | 0.727 (0.628 - 0.827) | 0.907 (0.820 - 0.994) | 0.933 (0.870 - 0.996) | 0.650 (0.529 - 0.771) | 0.818 | 0.132 | 0.7186585 |
| Model3 | 0.885 (0.827-0.944) | 0.833 (0.754-0.895) | 0.857 (0.779 - 0.935) | 0.791 (0.669 - 0.912) | 0.880 (0.806 - 0.954) | 0.756 (0.630 - 0.881) | 0.868 | 0.133 | 0.5788796 |
| Model4 | 0.870 (0.809-0.931) | 0.767 (0.681-0.839) | 0.662 (0.557 - 0.768) | 0.953 (0.891 - 1.000) | 0.962 (0.911 - 1.000) | 0.612 (0.495 - 0.729) | 0.785 | 0.141 | 0.7638413 |

Note: Model1: 2 mm peritumoral radiomics score model, Model2: 4 mm peritumoral radiomics score model, Model4: 6 mm peritumoral radiomics score model, Model4: 8 mm peritumoral radiomics score model. AUC: Area under the curve; CI: Confidence interval; PPV: Positive predictive value; NPV: Negative predictive value.

**Supplementary Table 2.** Prediction performance of Four Peritumoral Radiomics Models in the test set.

| Model | AUC (95%CI) | Accuracy (95%CI) | Sensitivity (95%CI) | Specificity (95%CI) | PPV (95%CI) | NPV (95%CI) | F1 score | Brier score | Cut-off |
| --- | --- | --- | --- | --- | --- | --- | --- | --- | --- |
|  |  |  |  |  |  |  |  |  |  |
| Model1 | 0.884 (0.782-0.986) | 0.882 (0.761-0.956) | 0.912 (0.816 - 1.000) | 0.824 (0.642 - 1.000) | 0.912 (0.816 - 1.000) | 0.824 (0.642 - 1.000) | 0.912 | 0.120 | 0.4622671 |
| Model2 | 0.870 (0.772-0.969) | 0.765 (0.625-0.872) | 0.706 (0.553 - 0.859) | 0.882 (0.729 - 1.000) | 0.923 (0.821 - 1.000) | 0.600 (0.408 - 0.792) | 0.800 | 0.138 | 0.7019815 |
| Model3 | 0.761 (0.621-0.901) | 0.804 (0.669-0.902) | 0.912 (0.816 - 1.000) | 0.588 (0.354 - 0.822) | 0.816 (0.693 - 0.939) | 0.769 (0.540 - 0.998) | 0.861 | 0.178 | 0.5641193 |
| Model4 | 0.824 (0.706-0.941) | 0.765 (0.625-0.872) | 0.706 (0.553 - 0.859) | 0.882 (0.729 - 1.000) | 0.923 (0.821 - 1.000) | 0.600 (0.408 - 0.792) | 0.800 | 0.159 | 0.7368929 |

Note: Model1: 2 mm peritumoral radiomics score model, Model2: 4 mm peritumoral radiomics score model, Model4: 6 mm peritumoral radiomics score model, Model4: 8 mm peritumoral radiomics score model. AUC: Area under the curve; CI: Confidence interval; PPV: Positive predictive value; NPV: Negative predictive value.

# 3 Supplementary **Figures**

**Supplementary Figure 1.** Peritumoral 0-2mm VOI Lasso regression results: **(A)**: Path plot of Lasso regression coefficients; **(B)**: Lasso regression performed using five-fold cross-validation to identify the optimal Log(λ) value.

**Supplementary Figure 2.** Peritumoral 0-4mm VOI Lasso regression results: **(A)**: Path plot of Lasso regression coefficients; **(B)**: Lasso regression performed using five-fold cross-validation to identify the optimal Log(λ) value.

**Supplementary Figure 3.** Peritumoral 0-6mm VOI Lasso regression results: **(A)**: Path plot of Lasso regression coefficients; **(B)**: Lasso regression performed using five-fold cross-validation to identify the optimal Log(λ) value.

**Supplementary Figure 4.** Peritumoral 0-8mm VOI Lasso regression results: **(A)**: Path plot of Lasso regression coefficients; **(B)**: Lasso regression performed using five-fold cross-validation to identify the optimal Log(λ) value.
